# Blooming as a New Nurse Amid Trials and Uncertainty: A Qualitative Content Analysis

**DOI:** 10.1155/jonm/5401225

**Published:** 2025-10-30

**Authors:** Yun-Jung Choi, Hae-In Namgung, Heewon Song, Na Rae Lee

**Affiliations:** ^1^Red Cross College of Nursing, Chung-Ang University, Seoul, Republic of Korea; ^2^Department of Nursing, Dongyang University, Yeonju, Republic of Korea

**Keywords:** adaptation, nurses, professional role, psychological, qualitative research, stress

## Abstract

**Background:**

New nurses often face a demand-ability mismatch during their transition to clinical practice, encountering a gap between the required skills and personal abilities. This mismatch contributes to negative emotions and transition shock, a phenomenon particularly pronounced among new nurses in Korea.

**Objective:**

This study aimed to explore and understand the transitional experiences of new nurses during their first year of clinical practice.

**Method:**

Novice nurses from Seoul, Korea, were recruited for this qualitative study. Data were collected through interviews and analyzed using content analysis.

**Results:**

New nurses reported facing uncertainty, overwhelming workloads, and insufficient support. These challenges were organized into: “doubt and self-perception,” “insurmountable tasks,” “striving for purpose,” and “finding solace through connections.” Despite these difficulties, their resilience emerged as a central theme, “blooming as a new nurse during trials and uncertainty.”

**Conclusion:**

New nurses experience significant uncertainty, self-doubt, and a gap between academic learning and the realities of clinical practice during their transition to practice. The findings provide a deeper understanding of their transition experiences and offer a foundation for strategies that support adaptation and workforce retention.

**Implications for Nursing Management:**

Nursing managers can translate these insights into best practices by adopting a dual approach—combining clinical transition programs, such as multipatient simulation, with stress management strategies—to strengthen adaptation, safeguard patient safety, and enhance organizational resilience.

## 1. Introduction

New nurse's first transition into a clinical setting is a challenging and pivotal period as they navigate their new role and develop a professional identity [1, 2]. While they initially approach this phase with excitement and confidence, many quickly realize they are unprepared for the realities of clinical practice [3]. Clinical environments demand that new nurses acquire essential knowledge rapidly and provide care that ensures patient safety and the safety of the hospital environment (e.g., infection control and fall prevention). However, new nurses often face significant barriers, including limited clinical knowledge, low confidence, high patient volumes, complex cases, and inadequate support systems [4]. This leads to a demand-ability mismatch—a gap between the skills required in the workplace and their current competencies [5].

This mismatch can elicit negative emotions—such as doubt, confusion, loss, anxiety, and fear—resulting in transition shock, a state of psychological distress experienced by nurses entering the clinical environment, driven by divergent expectations and workplace demands [3, 6, 7]. Several factors contribute to transition shock, including the assumption of new responsibilities and roles, insufficient social support, and knowledge gaps [3]. These encompass heavy practice workloads and challenges in clinical decision making and judgment; difficulties with shift adjustment and physical fatigue; limited guidance from preceptors and the absence of a supportive culture; and discrepancies between theoretical learning and practical application, as well as difficulties in managing diverse clinical symptoms [8–11]. Contributing factors include turnover intentions, dissatisfaction with department assignments, anxiety about medication administration, responsibility for managing multiple patients with varying conditions, and inadequate communication skills [12–14].

Transition shock among new nurses has been addressed internationally as an essential issue, with nurse residency programs (NRPs) and internship programs (INPs) in the United States, hospitals using orientation, and efforts to mitigate it in the United Kingdom, Australia, and other countries [4]. In Korea, preceptorship, mentoring, communication, and organizational socialization training are provided as programs for new nurses to adapt to clinical practice. The Korean version of the NRP program was recently proposed based on the U.S. NRP model. The Korean Ministry of Health and Welfare also implemented the “Clinical Nurse Educator Support Project” in 2019 to support the new nurses' adaptation at public hospitals, and by 2022, the project was expanded to cover 58 hospitals [15–17]. The aim of the project was to enhance the adaptability of new nurses by assigning nurse educators to provide systematic training and guidance. These transition support programs contribute not only to reducing turnover rates but also to enhancing patient safety, improving the quality of nursing care, and strengthening organizational resilience [18–20]. Therefore, nursing leaders need to systematically support new nurses' adaptation based on evidence derived from their transition experiences.

To become a nurse in Korea, one must obtain at least a four-year bachelor's degree in nursing and pass the national licensing exam administered by the Korea Health Personnel Licensing Examination Institute (KHPLEI) [21, 22]. This implies they got a standardized, high-level education. However, South Korea has a nurse-to-population ratio of 4.4 clinical nurses per 1000 people, which is only 45.4% of the Organisation for Economic Co-operation and Development (OECD) average of 9.7 [23], indicating that nurses in Korea face relatively high patient-to-nurse loads compared to their counterparts in OECD countries. These structural conditions further exacerbate the difficulties new nurses face during transition period. The turnover rate of new nurses in Korea is 47.7%, higher than that in the United States (22.9%) [24]. Moreover, in 2022, 57.4% of new nurses resigned within a year. The most common reason for resignation within 1 year was job maladjustment [25], indicating that new nurses in Korea struggle to overcome transition shock. This situation implies that support for new nurses is not merely a matter of individual adaptation but is also crucial at the managerial and organizational levels. Despite the high resignation and turnover rates of these nurses, research on novice nurses' transition experiences has focused on conceptualizing, analyzing influencing factors, and developing tools [26]. Qualitative research on new nurses' experiences during the transition period is relatively scarce. Therefore, there is a need for an in-depth exploration of new nurses' experiences as they transition into the clinical environment.

Qualitative analysis is an appropriate method for conducting this type of study, as it aims to provide an understanding and insight into the research phenomenon by examining the surface and latent meanings of the content [27, 28]. This method involves extracting meaningful statements from participants' descriptions of their experiences, generating general and abstract statements, categorizing them into themes, and describing the essential structures of these experiences. It is particularly suited for studying nurses' experiences as it respects participants' perspectives and interpretations of subjective experiences encountered during professional socialization. Accordingly, this study explored the experiences of new nurses using a qualitative approach to understand their transition into clinical settings. The research question was, “What are the experiences of new nurses during their first year as they transition to the clinical nursing environment?”

## 2. Method

### 2.1. Study Design

We utilized a qualitative design. Data were collected through interviews and analyzed using content analysis. The data were reported per Consolidated Criteria for Reporting Qualitative Research (COREQ) guidelines [29].

### 2.2. Participant Selection

Participants were recruited from semipublic and general hospitals located in Seoul, Korea. We disseminated a formal recruitment notice throughout the hospitals and used purposive sampling methods. Specifically, we sought nurses with less than 1 year of clinical practice. Eligibility criteria were nurses with less than 12 months of work experience after graduation. Recruitment continued until data saturation was reached. Three researchers (Na rae Lee, Hae-In Namgung, and Heewon Song) independently analyzed the interview data and agreed that saturation had been achieved after interviewing 23 new nurses, at which point no additional codes or themes emerged, and data collection was concluded.

### 2.3. Setting

Noncontact face-to-face video interviews were conducted using Zoom, allowing participants to choose a comfortable time. Only the researcher and participants were present during the interviews, with no nonparticipants in attendance. Data were collected between May 20, 2023, and July 1, 2023. All participants were new nurses working in Seoul, Korea, and the study was conducted within the Korean healthcare context.

### 2.4. Data Collection

Each interview lasted between 50 and 90 min. We used a semistructured interview guide. The main interview questions were “What emotions did you experience when transitioning to the clinical nursing environment?” and “What was your experience as a new nurse in transitioning to a clinical nursing environment?” Additional detailed questions included: “What is the most challenging aspect of being a new nurse?,” “As a new nurse, what have been your experiences with your coworkers?,” and “How do you perceive yourself on the ward?” Repeated interviews were not conducted. However, online video interviews may influence the interview process by altering social presence, eye contact perception, and impression management [30]. Two additional participants were recruited, and face-to-face interviews were conducted to ascertain the differences between them and ensure the validity of the interview content. The interviews were recorded with the participants' consent, and the content was transcribed. If any expression was unclear, the transcripts were sent to the participants for clarification.

### 2.5. Data Analysis

We used the qualitative content analysis method developed by Graneheim and Lundman [31]. Data were analyzed using a manual line-by-line coding process in Microsoft Excel. The coding process followed an iterative approach, with codes continuously reviewed and refined to develop themes. The process began with identifying meaning units through the repeated reading of interview transcripts relevant to study aims. Meaning units composed of words, phrases, or sentences sharing a central meaning were selected to reflect the text's themes comprehensively. In the next step, the meaning units are condensed, preserving their essence while simplifying their form. These condensed units were assigned codes that served as the foundation for the analysis. Based on their similarity, the codes were grouped into subcategories that were further abstracted into broader categories—a final review of the categories allowed for identifying themes, thereby revealing the text's latent meanings. The analysis was conducted iteratively, moving back and forth between the entire text and its parts to refine and validate the results. Three researchers (Na rae Lee, Hae-In Namgung, and Heewon Song) collaborated throughout the process and reviewed the coding and categorization to ensure consistency. Representative quotations have been included to enhance the credibility and clarity of the findings.

### 2.6. Internal Consistency

Three researchers (Na rae Lee, Hae-In Namgung, and Heewon Song) independently coded the interview transcripts and engaged in iterative discussions to reconcile discrepancies. Agreement on all codes and themes was reached through consensus, ensuring the credibility and consistency of the analytic process.

### 2.7. Rigor and Trustworthiness

Lincoln and Guba's [32] criteria of credibility, transferability, dependability, and confirmability were used to ensure qualitative rigor. Credibility was established by showing the study results to a nurse who had not participated but was working as a new nurse. To check transferability, new nurses who did not participate in the study compared the results with their own experiences to determine whether they were meaningful. Data dependability was ensured by following the proposed research procedures. Confirmability was addressed by consistently comparing the interview data between researchers to maintain neutrality throughout the study.

### 2.8. Ethical Consideration

This study was approved by the Institutional Review Board of a university in Korea (No. 1041078-20230331-HR-081). The purpose of the study, procedures, interview methods, recording, and data anonymization were explained to the participants, and verbal consent was obtained from those who expressed an interest in participating. The participants were also informed that they could withdraw from the study and would not be penalized. The possibility of the publication of these results is also explained. Data were stored on separate removable disks and anonymized.

## 3. Results

A total of 23 new nurses who comprehended the study's purpose and procedures and voluntarily agreed to participate were included in the study (Table 1).

The experiences of new nurses transitioning into a clinical nursing environment were analyzed and organized into a central theme supported by four main categories and their respective subcategories. The central theme, “blooming as a new nurse amid trials and uncertainty,” encapsulates their challenges and growth. The four main categories—lost in the shadows of doubt and self-perception, overwhelmed by insurmountable tasks, striving for stability and purpose, and finding solace through connections—reflect distinct facets of their transition experiences. Each category was further elaborated through subcategories, offering an understanding of the struggles, coping mechanisms, and support systems that shaped their adjustment to clinical practice (Table 2).

### 3.1. Lost in the Shadows of Doubt and Self-Perception

#### 3.1.1. Trapped in Endless Uncertainty

New nurses were trapped in a cycle of self-doubt and uncertainty during their transitions. Despite the passage of time, they struggled to perceive personal growth. They skipped meals and delayed bathroom breaks to complete their assigned tasks. However, feelings of failure persisted when they could not meet their expectations. This sense of failure transformed them into guilt and self-criticism, deepening their insecurity. These emotions diminished them and drove them deeper into their absence of uncertainty. Anxiety stemming from their uncertainties lingered beyond their shifts, making it difficult for them to detach from work even after leaving the hospital. Their daily lives became monotonous cycles of commuting between hospitals and homes, with thoughts of work dominating their personal time.

*“I worked hard for 8 hours, but nothing got done. When the next shift came and looked, the fluid remained, and did not notify a doctor about the patient's condition. Everything is paused from the previous shift to the next.”* (#20)

*“Ever since becoming a nurse, guilt and self-critical feelings have been overwhelming. I keep asking myself, ‘Why do I keep making mistakes?' Even when it was something my senior explained before, I worry about how to apply it correctly…These thoughts follow me home and do not go away. Phrases such as, ‘I already explained this before. Did you not study?' replay in my head, leaving me feeling small and discouraged.”* (#6)

#### 3.1.2. Seeing Myself as a New Nurse

New nurses trapped in a cycle of self-doubt often describe themselves negatively in the context of their roles in hospitals. They referred to themselves as having pain in the neck, feeling that, rather than contributing to the ward, they frequently required assistance from colleagues. They also perceived themselves as mechanical or robotic, completing an overwhelming workload without meaningful patient interaction or communication. This perception has led to confusion regarding nurses' professional roles and identities. Additionally, despite being in a position where slower performance was understandable, new nurses compared themselves to their more experienced colleagues, often labeling themselves slow, struggling, and pitiable. This self-perception highlights the significant internal challenges faced during the transition journey.

*“I kept thinking, ‘What have I been doing until this senior arrived? Why am I only causing trouble?' The stress was so overwhelming that I felt like I could not process anything about myself.”* (#17)

*“I focus on completing tasks as quickly as possible; before I know it, it is time to go home. Instead of feeling like I am truly caring for patients, I feel more like a robot performing tasks for an avatar.”* (#19)

### 3.2. Overwhelmed by Insurmountable Tasks

#### 3.2.1. Relentless and Unceasing Workload

When new nurses enter the workforce, there is often a disconnection between their school education and the role required in clinical nursing practice. Meanwhile, the clinical environment requires a constant and massive amount of research as new treatments are created and guidelines are constantly being updated. However, during an overwhelming workload, new nurses are expected to perform based on what they have learned, and they feel helpless when faced with so much they do not know and a never-ending to-do list.

*“Managing complaints, surgeries, and tests for 11 patients during regular shifts is exhausting. I am so busy I cannot even go to the bathroom or eat, yet the work keeps piling up on overwhelming days. No matter how hard I try, I still end up being a burden to the next shift, and that's when reality hits me hard.” (#18)*

#### 3.2.2. Working Beyond Common Sense

The workload alone did not overwhelm new nurses; relationships with colleagues and senior nurses also intensified the challenges of their transition experiences. Navigating interactions with overly critical or obsessive senior nurses became an additional strain as new nurses often felt compelled to monitor their moods constantly. The need to endure harsh verbal abuse and relentless negative feedback creates significant emotional barriers. These experiences weighed heavily on them, casting a shadow over their future and amplifying their difficulties adapting to the clinical environment.

*“When the room leaders say, ‘I told you, why could you not do it?' I feel pressured. When I feel pressured, I cannot consider other things. Because I concentrate on these emotions, I cannot listen to feedback well. I know this, but it is challenging to improve. Finally, this vicious cycle is repeated.*” (#22)

### 3.3. Striving for Stability and Purpose

#### 3.3.1. Exploring Stress Relief Strategies

New nurses create hope amid challenges, believing their daily struggles lay the foundation for personal and professional growth. They hold on to thinking they will see themselves as competent and experienced one day. By embracing the idea that complex individuals can be encountered anywhere, they can reinterpret criticism as an opportunity for learning and self-improvement. They also found ways to overcome hardships by discovering meaningful and rewarding aspects of their nursing practice. However, in interpersonal relationships, they often establish emotional boundaries and find comfort and solace.

*“I did not know how long I would stay. Therefore, I did not want to waste energy on relationships*—*it seemed exhausting. Instead, I kept my distance from others and focused solely on work. Keeping things strictly professional felt easier.”* (#1)

#### 3.3.2. Embracing Reality

New nurses process seniors' criticism differently and adapt their responses based on their individual personalities. Some chose to criticize their stride, while others sought to balance it by focusing on any praise they received. They realized that leaving their current position due to challenges would not necessarily secure a better future in another workplace. This recognition led them to stay in their roles, believing that, as the saying goes, “Time answers all.” They trusted that, with time, they would grow, and their difficulties would gradually diminish.

*“When I feel like quitting, I get overwhelmed. Therefore, I try not to think that way. Instead, I tell myself, ‘She scolded me because her environment is tough,' and try to understand.”* (#18)

*“On tough, busy days, I hold on to the hope that in a few months, I will have improved. I am just waiting for that day to come.”* (#4)

#### 3.3.3. Envisioning the Future as a Nurse

Despite the challenges in their transition journeys, these new nurses held aspirations for their futures. Their envisioned future was often framed as a way of repaying their gratitude, even amid the uncertainties they faced. Motivated by guilt stemming from their current inadequacies, they aspired to become nurses with a broader perspective capable of providing comprehensive patient care. They dreamed of evolving from dependent on new nurses to colleagues who could support senior nurses and assist their peers, embodying the roles of dependable and compassionate professionals.

*“I want to become the kind of nurse everyone turns to. Just like senior nurses are sought out when coworkers face challenges, I want to be someone who supports other nurses while also providing excellent care for patients!”* (#16)

### 3.4. Finding Solace Through Connections

#### 3.4.1. Emotional Support From Interpersonal Relationships

The weight of new nurses' transition journeys was influenced by interpersonal relationships, which either intensified or alleviated their burdens. They found solace and gratitude for the encouragement they received from patients and their families. Positive feedback from senior nurses and colleagues served as a source of energy, motivating them to move forward and persevere through challenges.

*“As I work, I ask myself if I am doing well. I believe it is anxiety. However, patients and teachers once told me, ‘You are doing well.' That is when I feel rewarded and believe I must work harder.”* (#22)

## 4. Discussion

We explored the transitional journey of new nurses, from nursing students to practicing professionals, using a qualitative approach. Findings revealed that, despite facing significant challenges, new nurses grew into their roles by developing strategies to navigate difficulties. This process was encapsulated in the theme “*blooming as a new nurse amid trials and uncertainty*.” Their journey offers important implications for nursing leaders at the individual, organizational, and managerial levels.

Participants experienced doubts about their competencies and values due to the pressure and persistent anxiety about performing their roles, as well as frequent self-doubt and uncertainty, and did not recognize their sense of professional growth. This internal conflict can be interpreted as a result of the complex interaction of structural and psychological burdens experienced during the initial clinical experience. According to previous studies, new nurses experience self-doubt and psychological exhaustion during the transition period due to the gap between their professional role identity and actual performance, lack of support, and role ambiguity [33–35], which can lead to anxiety, decreased work engagement, and increased intention to leave [36]. Moreover, high workloads and insufficient support from senior colleagues increase psychological and physical fatigue, leading to poor physical health, negative mental health outcomes such as depression, and decreased job satisfaction [37, 38]. These challenges highlight that transition shock is not merely an individual problem but a structural issue that requires nursing leaders to recognize the psychological burdens faced by novice nurses and to provide targeted mentoring and role clarification as part of early adaptation support.

In order to cope with these pressures, new nurses sought their own stability and purpose through their own stress-relief strategies, such as exercise and hobbies, while also gaining confidence from completing tasks and supporting colleagues. Others sought solace through connections, finding comfort in peer empathy and encouragement from patients and families, findings consistent with previous studies [39]. These findings imply that while personal coping strategies are important, they are insufficient alone, and nursing leaders should acknowledge these efforts and integrate them into organizational support systems that promote resilience.

With the increasing entry of Generation Z into the workforce, most participants in this study belonged to this generation and exhibited distinctive characteristics in their relationships with colleagues. Specifically, they perceived work as self-actualization and new challenges and were able to separate their work from their personal lives [40]. While previous studies have shown that nurses' overloaded workloads make it difficult to separate work and home, negatively affecting their personal lives [41], this study found that separating work and personal lives helped them control their stress and positively affected their adaptation to the clinical environment. However, in a nursing culture where communication between colleagues is essential during handovers, the separation strategy of new nurses may lead to a breakdown in communication with mid-career nurses. These generational characteristics suggest that nurse managers must recognize diversity in work values and communication styles and develop strategies that foster collaboration across generations to enhance teamwork.

Building on these individual-level insights, it is also essential to examine how organizations can support. One of the best ways to mitigate transition shock is to provide sufficient orientation in hospitals [42–44]. The new nurses in this study underwent training programs of varying length and content depending on the hospital, ranging from as little as 2 weeks to as much as 2 months. Regardless of duration, most participants perceived that the orientation was insufficient. Since such programs primarily operate in public and tertiary general hospitals, their applicability is limited because of the limited staffing [45]. Therefore, it is necessary to provide new simulation education methods in addition to existing classes in schools and hospitals. Nursing schools worldwide focus on reducing the gap between clinical and school education and improving learning capabilities by designating simulation education as a required course [46]. However, on-campus simulation education methods are limited to a single model and situation. Previous studies have confirmed the efficacy of multipatient simulation [47–49]. Therefore, patient care in multiple situations is necessary before transitioning to the clinical environment through multipatient simulation. These findings highlight the need for institutional and educational interventions that complement individual coping and also provide the basis for managerial strategies to sustain the nursing workforce.

At a broader level, the findings of this study call attention to the responsibility of nursing managers and clinical leaders to translate evidence from new nurses' transition experiences into best practices. Our findings emphasize the challenges of the new nurses. The gap between the academic environment and clinical practice, together with feelings of inadequacy and disappointment, makes clinical adjustment particularly difficult. Given these challenges, a dual approach is required. Stress management programs are essential to help new nurses cope with the emotional and physical demands of their roles, reducing psychological distress, improving retention, and preparing them to deliver safe care. At the same time, comprehensive clinical adaptation education, such as multipatient simulations replicating real practice environments, is vital to building confidence and clinical readiness. These two strategies need to be integrated as complementary interventions so that new nurses become not only technically competent but also resilient and psychologically prepared for practice. This dual approach provides the basis for nursing managers and clinical leaders to develop best practices that ensure patient safety, increase new nurses' retention, and strengthen organizational resilience.

## 5. Limitations

There is a cultural context limitation. Since we only explored new nurses' transition experiences in Seoul, Korea, findings may not be applicable to new nurses in other geographical and cultural settings. Thus, future studies should explore different cultural settings or use the meta-synthesis method to provide a broader understanding of new nurses' transition experiences. Furthermore, based on the necessity of the dual approach emphasized in this study, research on the development and effectiveness of a transition program is needed.

## 6. Conclusion

Transition shock experienced by new nurses in Korea revealed challenges such as uncertainty, self-doubt, and the gap between academic preparation and clinical realities. Although existing support programs are helpful, they are often insufficient, contributing to high resignation rates and quality of care concerns. These findings highlight the need for a dual approach for clinical transition programs, such as case-based clinical simulations that involve multiple patient scenarios and stress management strategies, in better supporting new nurses in overcoming transition shock and adapting to professional practice. Future efforts need to integrate these strategies into organizational policies to support sustainable workforce resilience.

## Figures and Tables

**Table 1:** Characteristics of participants.

Participants	Age (years)	Experience (month)	Sex	Work environment
Nurse 1	24	8	Female	General hospital
Nurse 2	33	10		
Nurse 3	27	6		
Nurse 4	24	8		
Nurse 5	23	11		
Nurse 6	24	11		
Nurse 7	27	9		
Nurse 8	23	3		
Nurse 9	24	3		
Nurse 10	23	3		
Nurse 11	25	3		
Nurse 12	25	3		
Nurse 13	25	1		
Nurse 14	23	11		

Nurse 15	35	3	Female	Semipublic hospital
Nurse 16	24	11		
Nurse 17	25	5		
Nurse 18	23	5		
Nurse 19	23	5		
Nurse 20	24	11		
Nurse 21	23	11		
Nurse 22	24	11		
Nurse 23	25	3	Male	

**Average**	**25.04**	**7**		

**Table 2:** Theme derived from the new nurses' transition experiences.

Theme		
Blooming as a new nurse amid trials and uncertainty		
Categories	Subcategories	Codes
Lost in the shadows of doubt and self-perception	Trapped in endless uncertainty	• Feeling unsure about my progress
		• A shrunken and intimidated self
		• Bound by home and hospital
	Seeing myself as a new nurse	• A pain in the neck
		• Mechanical nurse
		• Struggler

Overwhelmed by insurmountable tasks	Relentless and unceasing workload	• Overwhelming influx of tasks
		• Poor working conditions
		• The gap between school education and practice
	Working beyond common sense	• Working with sensitive coworkers
		• Negative feedback as an obstacle to stepping forward

Striving for stability and purpose	Exploring stress relief strategies	• Easing my mind
		• Chasing the bluebird as a nurse
		• Setting emotional boundaries in relationships
	Embracing reality	• Faith that time will heal
		• Choosing now over the unknown
		• Accepting seniors' rebuke my way
	Envisioning the future as a nurse	• A nurse with a broad perspective
		• A supportive nurse who can assist others

Finding solace through connections	Emotional support from interpersonal relationships	• Encouragement of patients and caregivers
		• Supportive seniors and fellows
		• Positive feedback as the accelerator goes step forward

## Data Availability

Data are available upon reasonable request. Please contact the corresponding author for data requests.
